# 65 First francophone e-learning of pediatric rheumatology in Africa: the trainers’ opinion

**DOI:** 10.1093/rheumatology/keac496.061

**Published:** 2022-10-07

**Authors:** Cherif Ines, Ferjani Hanene, Khamessi Ichrak, Maatallah Kaouther, Guedri Rahma, Miladi Saoussen, Ben Khaled Monia, Fazaa Alia, Jallouli Manel, Ben Abdelghani Kaouther, Khemiri Monia, Fitouri Zohra, Hamdi Wafa

**Affiliations:** 1 Med Kassab Institute of Orthopedics, Rheumatology Department, Manouba, Tunisia; 2 Bechir Hamza Children Hospital, Beb, Tunisia, Saadoun; 3 Mongi Slim Hospital, Rheumatology Department, La, Tunisia, Marsa; 4 National Bone Marrow Transplant Centre, Hematology Department, Beb, Tunisia, Saasoun

## Abstract

**Background:**

Pediatric Rheumatology is currently gaining importance across Africa. Initiatives such as the creation of the paediatric society of the African league against rheumatism (PAFLAR) proved the efforts made to bridge the insufficiency. In the same line, training in pediatric rheumatology remains the main challenge of this orphan subspecialty. For this purpose, a post-graduate certification in Pediatric Rheumatology was created in 2021 in the Faculty of Medicine of Tunis—Tunis-El Manar University. It is the first online certification dedicated to French speakers in Africa. The certification is entitled “musculoskeletal diseases of children and adolescents”.

**Objective:**

To assess the learners' perceptions and views of this certification.

**Methods:**

We established an online survey targeting the participants. The questionnaire was divided into three main sections: the first section examined the participant characteristics: their specialty, the use of the Moodle platform of the Virtual University of Tunis (UVT), their previous participation in an online post-graduation course, etc.). The second section focused on platform acceptability. The third and final section evaluated the content of the course. The Likert scale was used in most questions.

**Results:**

Fifteen participants responded to the survey. Among them, one is specialized in family medicine, 1 in orthopedics, 5 in pediatrics, and 8 in rheumatology. Fifty-three percent have never used the platform of the virtual university of Tunis (UVT) before, and 26.7% have never attended an online course. Most participants found the platform easy to access and easy to use with a mean Likert scale of 2.2 and 2.13, respectively. In the third section of the questionnaire, the course met the expectations of the candidates with a mean Likert scale of 2.1. Most of them found the post-test useful to their learning experience, and only two found it to be the opposite. This can be explained by the pressure that it may generate. Eighty-six percent of the candidates had an interactive experience with the educator and enjoyed the interactions and the discussions between the participants. Most participants were pleased with the seminar organization and with the number of h devoted to each seminar (mean Likert Scale = 2.1). Eighty-six percent of the participants have found that the course improved their medical practice and 93.3% confessed that they were satisfied with the online nature of the certification and did not wish it to be physical.

**Conclusion:**

Overall, the post-graduate “musculoskeletal diseases of children and adolescents” course was beneficial and enriching/rewarding for the participants.

Developing this training program helps to promote pediatric rheumatology in Africa and to provide children with rheumatic diseases access to healthcare.

